# Defining service catchment areas in low-resource settings

**DOI:** 10.1136/bmjgh-2021-006381

**Published:** 2021-07-22

**Authors:** Peter M Macharia, Nicolas Ray, Emanuele Giorgi, Emelda A Okiro, Robert W Snow

**Affiliations:** 1 Centre for Health Informatics, Computing, and Statistics, Lancaster Medical School, Lancaster University, Lancaster, UK; 2 Population Health Unit, KEMRI-Wellcome Trust Research Programme, Nairobi, Kenya; 3 GeoHealth group, Institute of Global Health, University of Geneva, Geneva, Switzerland; 4 Institute for Environmental Sciences, University of Geneva, Geneva, Switzerland; 5 Centre for Tropical Medicine and Global Health, Nuffield Department of Medicine, University of Oxford, Oxford, UK

**Keywords:** epidemiology, health policy, indices of health and disease and standardisation of rates, health services research, health systems

**Summary box**

- Defining an accurate, representative service catchment area is important for computing population denominators for disease mapping and efficient public planning, including health, education and social care.
- The growth in population settlement modelling techniques and provision of geocoded service databases has fuelled an increase in local and regional service access mapping to examine coverage and equity in much of sub-Saharan African countries.
- However, metrics of service access and catchments are often implemented based on convenience, disregarding the implications on accuracy of the catchment population, complexities of service use and the likely implications for public service planning.
- Lack of high spatial resolution geolocated data on residential locations of the service users has led to the use of rudimentary, inexact approaches to complex processes that define service catchment areas and should be used with caution.
- The improved collection of residential addresses of service users and service providers has increased the ability to develop new innovative models of service catchment.
- Improved data availability and data sharing must be accompanied by better models of service use.
- In this commentary, we revisit the issue by considering common approaches, key issues and best practices in defining a reliable service catchment area.
- We hope this will lead to further granular studies to populate and compare methods to improve the definition of service catchment areas in sub-Saharan Africa, ultimately improving efficiencies and equity in service use and more reliable interpretations of routine service use data.

## Introduction

An accurate service catchment area—a geographical area delineated around a service point (such as a health facility or a school) describing the population that uses its services^1^—is important for robust and reliable estimation of population denominator used in planning, estimating commodity needs and mapping applications. In the literature, approaches used to define a service catchment area range from simple to complex approaches and are often presented as population to service ratios, use of administrative boundaries, proximity-based metrics and models accounting for utilisation rates. However, the implementation and choice of an approach are often based on convenience, disregarding the implications on accuracy of the catchment population, complexities of service use and the likely implications for planning. In this commentary, we highlight the common approaches, key issues and best practices in defining a reliable and representative service catchment area. We hope to create a dialogue within the geospatial community and allied stakeholders to evolve best-practice standards in defining service catchment areas for efficient and equitable planning.

The commentary is structured into four sections. We discuss the need for recording and geolocating user addresses followed by a summary of approaches used to define service catchment areas and conclude with the key characteristics of an ideal service catchment, best practices and key issues that require addressing.

## Need for recording and geolocating service user addresses

The definition of a reliable service catchment area for health, education, social care and other local public services depends on the availability of spatially positioned residential addresses of those who seek services. In high-income countries, residential addresses (such as zip code, house number and street name) are part of well-defined registers that can be used to define service catchment areas. Conversely, most of sub-Saharan Africa (SSA) do not have the luxury of well-defined addresses or routine logging and geocoding of villages/estates of those seeking services.^2 3^ In cases where such data are available in SSA, there is heterogeneity between and within countries. For example, within health and demographic surveillance sites, addresses of households are well maintained. The absence of reliably defined service catchment areas challenges robust disease mapping, equitable provision of vaccines (eg, childhood immunisations), other preventive health commodities (such as bed-nets), and acute care, school needs, social care and the accurate compilation of health data. This is because such applications rely on a catchment population denominator, which is in turn derived by a spatial overlay of population density maps and the geographical extent of a service catchment area.^4–7^

Across SSA, population settlement and sites of service provision are often not mapped on a regular basis nor are always available at small administrative units for efficient planning. Disaggregating low-resolution census data to presumed high-resolution settlement patterns guided by satellite imagery and building footprints has increased in sophistication in recent years.^8^ The locations of schools, health facilities and other amenities have improved with crowd-sourced Global Positioning Systems initiatives, participatory mapping, improved national gazetteers, high-resolution satellite imagery and demand for such data sets.^3^ However, linking population to service delivery remains crude and ignores the complexities of service demand and/or supply mainly due to a lack of geocoded data on where the users of services reside in SSA.

## Modest approaches used to define service catchment areas

**Figure 1:**
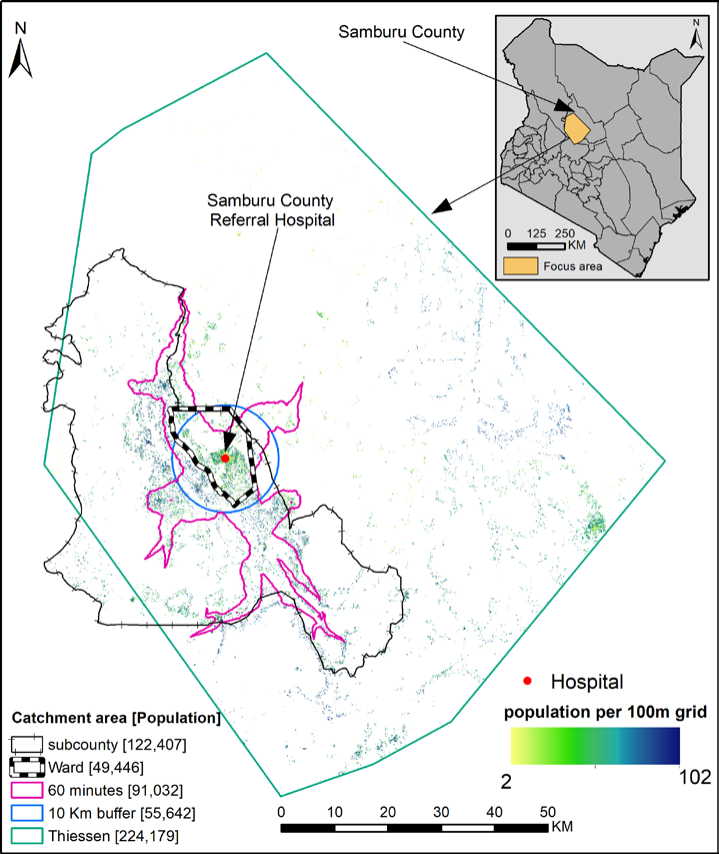
An illustration of several approaches used to define service catchment area, administrative unit (ward or subcounty), straight-line distance (10 km buffer and Thiessen polygon) and travel time (area within 60 min of a hospital). Illustration is based on 622 COVID-19 vaccination posts (https://medical.unon.org/node/169) approved in Kenya. If vaccine allocation was based on one of these catchment areas, there would be mismatches between those who attend a facility and the estimated population denominator in 2021^8^ across methods due to lack of geolocated residential data of care seekers at a particular post. The top right corner shows the illustrated region within Samburu County, Kenya.

Population-to-service ratios are the most common metrics, ranging from wide subnational units to national estimates for international ranking. These metrics merely require an estimate of population size from national censuses and the number of facilities within a census-defined polygon. Smaller, subnational polygons can be used, for example, subcounties, wards, municipalities or parishes (figure 1), where service provision is decided by local health and education authorities. However, people residing along administrative boundaries will likely access the most proximal service which might be in the neighbouring administrative unit. Similarly, people in need of a particular service far away from the border might be attracted to a particular service point in a neighbouring unit due to preferred, desirable characteristics. For example, women bypassed the nearest facility in Tanzania,^9^ Ghana,^10^ Kenya,^11^ Mozambique, India and Pakistan.^12^ As a result, it is common practice to have childhood immunisation coverage from routine data greater than 100% in administrative units that attract many people from neighbouring administrative units.^13 14^ This is likely due to a challenge of assigning population denominators to an administrative area instead of a well-defined service catchment area based on the actual users of a particular service.

Where both the population (whether actual or modelled) and amenity locations (geocoded with a longitude and latitude) are available at high resolution and accuracy,^8 15^ service catchment areas can be empirically modelled. The most common and rudimentary approach is the use of an arbitrary radius (buffer) around a service point, for example, a 10 km buffer. The radius of the buffer can be variable depending on the capacity of the provider, type of services offered and the mode of transport used to access the service (figure 1). Related to the buffer is Thiessen polygon, where each catchment area incorporates all points that are closer to a given service provider than any other provider (figure 1). These two approaches rely on straight-line distance, which does not reflect actual distance and/or time travelled, which are in turn impacted by topography, land use/cover, road network, transport modes and associated transport speeds. Furthermore, service points are also weighted equally despite differences in the capacity and size.

## Improved approaches to defining service catchment areas

The complexity of physical access, choice and demand characteristics can be tackled with more complex models of service use. *Gravity models* assume that the flow of people from residential areas to service providers is proportional to the demand for services and inversely proportional to physical access.^16^ The demand for services is defined by morbidity, age, and social structure and the capacity of a facility.^16^ To be more realistic, this physical access can be estimated by modelling the travel time to the nearest provider, by accounting for travel factors and barriers mainly through *cost distance algorithms* and *network analysis*.^17^ The travel time is then binned to define a service catchment area^1^ based on either an arbitrary time threshold (figure 1) or known cut-offs, where available (eg, 2 hours for obstetrical complications). A range of software including AccessMod,^18^ ArcMap (Esri, Redlands, CA, USA, 2021) and QGIS (QGIS.org, 2021. QGIS Association. QGIS Geographic Information System) can be used to estimate travel times and corresponding catchment areas.

The use of travel time is appealing, but it requires expert knowledge to implement. It also assumes travel time to the nearest facility, which is not always the case and often does not account for competition and user preferences.^12^ Furthermore, catchment boundaries might be highly permeable when arbitrarily defined thresholds are applied.^4^ The issue of arbitrarily defined time threshold can be minimised by combining travel time with utilisation rates typically from sample household survey data,^19^ to inform a reasonable threshold via the inflection point of a decay curve.^20^ The gravity models can be adapted with additional information and sophisticated models to account for competition between service points, variation in capacity, available equipment, quality and affordability of the service provided.^21^

## Ideal service catchment area and best practices

An ideal service catchment area should meet a minimum set of conditions from both the supply and demand side: (a) capture a significant proportion of its activities, (b) exclude areas whose activity contribution is due to random variation (eg, service users who are not usual residents such as visitors), (c) account for geography (physical access and its barriers), (d) differentiate across specialty types (eg, vaccination sites, tuberculosis detection, snake antivenom availability, or primary and tertiary educational levels) and capacities (affordability, size, staffing and equipment), (f) competition from the network of neighbouring providers and (g) seasonal changes due to weather or population flows.^22 23^

The use of models to define a catchment area can, however, be bypassed if data needed to define an ideal catchment were available, enabling the use of simpler geospatial information systems techniques. Therefore, defining a representative catchment is premised on the availability of geocoded data at high spatial and/or temporal resolution, reflecting residential location of service users, service points, utilisation rates, service use and factors that affect travel and behavioural data on service choice. Future ambitions to define service catchment areas should be aligned to several key considerations listed in table 1.

**Table 1:** Best practices and ambitions associated with defining reliable, accurate and representative service catchment areas for public services such as healthcare, education and social services

Category	Ambitions and best practices
Data collection	Improving collection and geocoding of residential address (village/estate) data from service users by healthcare providers, educational institutions, local governments and national statistical agencies. This will enhance the definition of service catchment areas for effective planning. High-resolution population density maps and databases of road network, land use/cover, travel barriers, care-seeking behaviour, modes of transport and travel speeds also need a careful curation.
Data and software sharing	Important data sets to define service catchment areas should no longer be kept in silos. The culture of making open-access data analytical models and software should improve across researchers and organisations in SSA. With increasing model sophistication, there is a need for software that can easily be used to define realistic service areas especially for planners.
Community	Building a community of researchers, sharing best practices, identifying difference between services, different diseases, service interruptions (eg, COVID-19 or natural disasters), ecological contexts and demography will be useful.
Service use	With a growth in availability of geocoded data and spatial epidemiologists across SSA, there is a need for an increased investment in research aimed at mapping higher resolution data on service use. Studies should also consider different forms of service access such as vaccination, healthcare, education and social care.
Disease mapping	The use of spatial statistics to map diseases, health outcomes, and demographic and socioeconomic indicators has witnessed huge advancements. However, the use of data from routine services (such as disease registries) together with reliably defined catchment areas requires more attention and quantifying the role played by different approaches and their impact on the mapped quantities.
Sensitivity	Where modelling must be conducted due to inadequate data, authors should test the sensitivity and uncertainty of several models that are used to define a service area. Comparisons will tease out if there any gains in using complex geospatial approaches in lieu of simpler approaches (more accessible to non-experts) to define service areas. There is also a need to recognise limitations such as bypassing the nearest provider due to personal preferences, quality and capacity when results are being interpreted.

SSA, sub-Saharan Africa.

In conclusion, service choice will always depend on a complex array of social factors, quality (perceived or actual), previous experience, affordability and physical accessibility encompassing both the supply and the demand side.^24^ Such factors dictate how often lower level health facilities are by-passed, or the choice of an educational institution or social care programmes.^25 26^ The availability of well-curated and regularly updated service utilisation data and locations of service users will be important in accounting for such unique characteristics and defining reliable service catchment areas going forward.
